# Activation of NRF2 by p62 and proteasome reduction in sphere-forming breast carcinoma cells

**DOI:** 10.18632/oncotarget.3047

**Published:** 2015-02-27

**Authors:** In-geun Ryoo, Bo-hyun Choi, Mi-Kyoung Kwak

**Affiliations:** ^1^ College of Pharmacy, The Catholic University of Korea, Bucheon, Gyeonggi-do, Republic of Korea

**Keywords:** mammospheres, cancer stem cell, resistance, NRF2, p62

## Abstract

Cancer stem cells (CSCs) express high levels of drug efflux transporters and antioxidant genes, and are therefore believed to be responsible for cancer recurrence following chemo/radiotherapy intervention. In this study, we investigated the role of NF-E2-related factor 2 (NRF2), a master regulator of antioxidant gene expression, in the growth and stress resistance of CSC-enriched mammosphere. The MCF7 mammospheres expressed significantly higher levels of the NRF2 protein and target gene expression compared to the monolayer. As underlying mechanisms, we observed that proteolytic activity and expression of the proteasome catalytic subunits were decreased in the mammospheres. Additionally, mammospheres retained a high level of p62 and the silencing of *p62* was observed to attenuate NRF2 activation. NRF2 increase was confirmed in sphere-cultures of the colon and ovarian cancer cells. The functional implication of NRF2 was demonstrated in *NRF2*-knockdown mammospheres. *NRF2*-silenced mammospheres demonstrated increased cell death and retarded sphere growth as a result of target gene repression. Moreover, unlike the control mammospheres, *NRF2*-knockdown mammospheres did not develop anticancer drug resistance. Collectively, these results indicated that altered proteasome function and p62 expression caused NRF2 activation in CSC-enriched mammospheres. In addition, NRF2 appeared to play a role in CSC survival and anticancer drug resistance.

## INTRODUCTION

The cancer stem cell (CSC) concept was first introduced in a study by Bonnet et al. [1], which demonstrated that a subpopulation of acute myeloid leukemia (AML) cells was capable of tumor initiation in non-obese diabetic mice. Ignatova et al. [2] reported that the human cortical glial tumors contain tumor stem-like cells. After these reports, CSCs were identified and characterized in various human tumors, including the cancers of the brain, breast, and colon [3, 4]. CSCs and normal stem cells share many properties such as self-renewal and differentiation capacities. Additionally, normal stem cells and CSCs are controlled by common signaling pathways such as Notch-1 and Wnt signaling for maintenance of the stem cell linage [5, 6]. The existence of CSCs is considered as the main cause of tumor relapse after chemo/radiotherapy in the clinic [7, 8]. The resistance can be explained by several molecular characteristics. CSCs present reduced levels of reactive oxygen species (ROS) and higher expression levels of drug efflux transporters than non-stem cells [9]. These changes can help in facilitating the extracellular efflux of anticancer drugs and enhance the protection against ROS damage, which results in chemo/radioresistance.

Since Reynold et al. [10] reported a neurosphere culture system using neuronal cells from the striatum of mouse brain, nonadherent sphere culture has been applied to assess self-renewal and pluripotency of CSCs *in vitro*. In anchorage-independent conditions, normal epithelial cells cannot survive because the loss of the cell-matrix interaction triggers anoikis, a form of programmed cell death [11]. On the other hand, undifferentiated cells such as stem cells or early progenitor cells can survive and form spheres in the same conditions [10, 12]. Dontu and colleagues developed a 3-dimensional nonadherent culture system using mammary epithelial cells from normal breast tissues. This system is called a mammosphere culture system [13]. They also found that these mammospheres are enriched in stem and early progenitor cells that can differentiate into epithelial linages. Similarly, more than 95% of the cells in mammospheres from breast cancer cell lines and primary breast tumors were CD44-positive and CD24-negative linages, which indicates that most of the cells in mammospheres are breast CSCs [14]. Hence, the mammosphere culture system has been utilized to screen or test anticancer drugs against breast CSCs along with the induced pluripotent stem cell [15, 16].

NF-E2-related factor 2 (NRF2) is a transcription factor that protects cells against oxidative damage by up-regulating the expression of various genes encoding detoxifying/antioxidant proteins, including NAD(P)H quinine oxidoreductase (*NQO-1*), heme oxygenase-1 (*HO-1*), glutathione (GSH) generation enzymes, and GSH peroxidase (*GPx*) as well as drug efflux transporters [17, 18]. Under normal conditions, NRF2 remains inactive through the formation of a complex with Kelch-like ECH-associated protein 1 (KEAP1), and this complex leads to the Cullin 3 (CUL3)-based E3 ligase-mediated ubiquitination and proteasome-dependent degradation of NRF2 [19, 20]. Canonical NRF2 activators such as sulforaphane induce the modification of KEAP1 cysteine residues, leading to the blockage of KEAP1/CUL3-mediated NRF2 degradation, and consequent transcription of the antioxidant response element (ARE)-bearing genes [21, 22]. In addition to this canonical pathway, several endogenous proteins such as p62 and p21 could increase NRF2 activity as a non-canonical way [23–25]. Particularly, p62, which is known as a linker molecule between ubiquitinated proteins and the autophagy system, was found to bind to specific residues of KEAP1 and, thereby, induce the dissociation of the NRF2-KEAP1 complex [24–26]. Although the essential role of NRF2 in normal cell redox homeostasis has been firmly established, a number of studies suggest that a high level of NRF2 in cancer cells results in growth enhancement and chemoresistance (reviewed by Hayes et al. [17]). In agreement with these data, our previous reports demonstrated that *NRF2* silencing suppressed tumor growth and chemoresistance in colon cancer cells [27] and ovarian cancer cells [28]. Moreover, recent evidence started to indicate the association of NRF2 with CSCs function. In neural stem/progenitor cells, NRF2 overexpression enhanced neurospheres formation and neuronal differentiation [29]. Zhu et al. [30] demonstrated that *NRF2* knockdown in primary human glioblastoma cells decreased self-renewal capacity of glioma stem cells (GSCs).

Since NRF2 governs the expression of genes encoding detoxifying/antioxidant proteins and drug efflux transporters, it can be hypothesized that chemoresistance and low ROS levels in CSCs can be mediated by NRF2 signaling. Herein, we investigated the role of NRF2 signaling in CSC survival and resistance by using a CSC-enriched MCF7 mammosphere culture system. We observed NRF2 signaling activation in MCF7 mammospheres as well as other type of cancer spheres, and suggested its underlying molecular mechanisms by demonstrating the diminished proteasome activity and elevated p62 level. Finally, NRF2-mediated up-regulation of detoxifying/antioxidant genes and efflux transporters was shown to be responsible for enhanced mammosphere growth and anticancer drug resistance.

## RESULTS

### Up-regulation of drug transporters and detoxifying/antioxidant genes in MCF7 mammospheres

**Figure 1:**
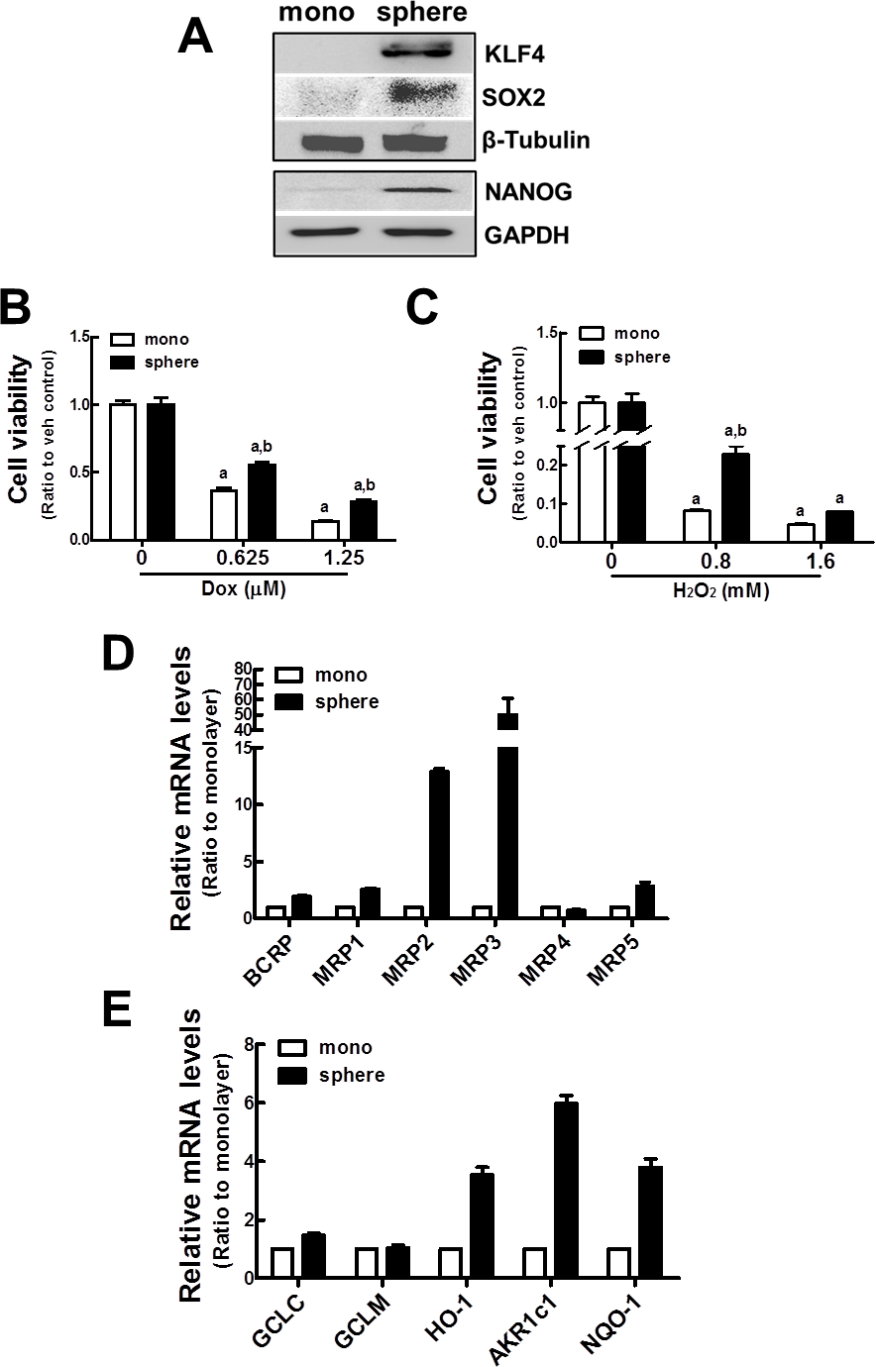
Up-regulation of ABC transporters and detoxifying/antioxidant genes in MCF7 mammospheres **(A)** MCF7 cells were grown as mammospheres for 7 d, and KLF4, SOX2, and NANOG protein levels were determined by western blot analysis.**(B–C)** Cell viability was monitored after doxorubicin (Dox, B) or hydrogen peroxide (H_2_O_2_, C) incubation for 72 h in MCF7 monolayers and mammospheres. Values represent the mean ± SE from eight sampled wells. ^a^*P* < 0.05 compared with non-treated group. ^b^*P* < 0.05 compared with monolayer. **(D)** *BCRP* and *MRPs* (*MRP1~5*) transcript levels were assessed in monolayers and mammospheres by real-time PCR for relative quantification. **(E)** *GCLC, GCLM, HO-1, AKR1c1*, and *NQO-1* transcript levels were assessed by real-time PCR. Values represent the mean ± SE from 3 experiments.

In order to establish an experimental system of tumor sphere formation, the human breast carcinoma MCF7 cell line was grown in an ultralow attachment plate with the sphere culture medium for 7 d. Then, the expression of CSCs markers such as KLF4, NANOG, and SOX2 was monitored by immunoblot analysis. As previously reported, protein levels of these CSCs markers were higher in mammospheres than in monolayer cultured MCF7 cells (Figure 1A). Specific cell surface markers are used to characterize CSCs. In breast CSCs, expression of CD44^+^/CD24^−^ has been associated with CSCs characteristics [31]. In our mammosphere system, the level of the *CD44* transcript was 4 times higher in mammospheres than in the MCF7 monolayer (Supplementary Figure S1). Additionally, cell population with expression of CD44^+^/CD24^−^ was more than 90% in mammosphere MCF7 (Supplementary Figure S2). These confirm that MCF7 mammospheres are a CSCs-enriched experimental system. Then, we examined whether MCF7 mammospheres acquired resistance to the anticancer drug, doxorubicin, and the oxidative stressor, hydrogen peroxide (H_2_O_2_). When the cells were incubated with doxorubicin (0.625 and 1.25 μM) for 24 h, the number of viable cells was higher in the mammosphere than in the monolayers. Thirty six percent of cells were viable in monolayers, while 55% were viable in the mammospheres after 0.625 μM doxorubicin (Figure 1B). Similarly, MCF7 mammospheres were relatively more resistant to H_2_O_2_ treatment than MCF7 monolayers (Figure 1C). These results confirm that the MCF7 mammosphere system possesses the core features of CSCs, including stemness gene expression and anticancer drug resistance.

Next, the expression of drug transporter genes was investigated in MCF7 CSCs by real-time reverse transcriptase (RT)-polymerase chain reaction (PCR). Levels of multidrug resistance proteins (*MRPs*; *ABCCs*) and breast cancer resistance protein (*BCRP*; *ABCG2*) were higher in mammospheres. In particular, *MRP2* and *MRP3* transcript levels were elevated by 13-fold and 50-fold, respectively, compared to MCF7 monolayers (Figure 1D). In addition, levels of γ-glutamate cysteine ligase (*GCLC*), *HO-1*, aldo-keto reductase 1c1 (*AKR1c1*), and *NQO-1* were elevated by 1.5–, 3.5–, 6.0–, and 3.8–fold, respectively, in MCF7 mammospheres when compared to MCF7 monolayers (Figure 1E). Taken together, these results suggest that efflux transporters and detoxifying/antioxidant genes were elevated in MCF7 mammospheres.

### Activation of NRF2 signaling in MCF7 mammospheres

**Figure 2:**
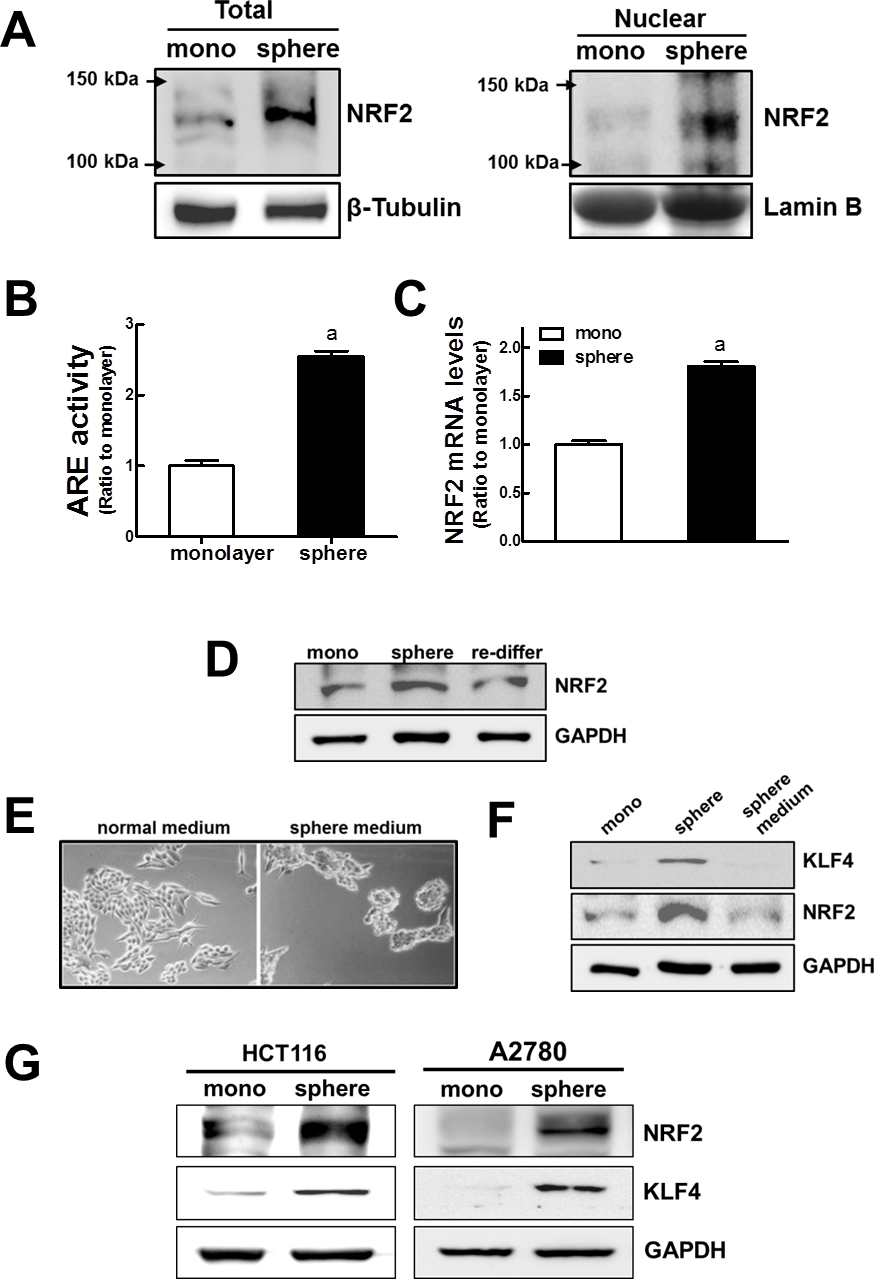
Activation of NRF2 signaling in MCF7 mammospheres **(A)** Total and nuclear NRF2 protein levels were determined in monolayers and mammospheres by western blot analysis. **(B)** NRF2 transcription activity was monitored using a *NQO-1* ARE-driven luciferase reporter. **(C)** *NRF2* transcript levels were assessed in monolayers and mammospheres by real-time PCR. Values represent the mean ± SE from 3 experiments. ^a^*P* < 0.05 compared with MCF7 monolayer. **(D)** NRF2 protein levels were determined in MCF7 monolayers, mammospheres, and re-differentiated MCF7 mammospheres by western blot analysis. **(E)** MCF7 cells were cultured in a normal adherent plate under normal medium or sphere medium for 3 d and microscopic observation was performed. **(F)** In these conditions, NRF2 and KLF4 protein levels were assessed by western blot analysis. **(G)** Levels of NRF2 and KLF4 were determined in sphere-cultured HCT116 and A2780 by western blot analysis.

Based on the elevated expression of detoxifying/antioxidant genes and drug efflux transporters, we investigated whether the NRF2 signaling pathway was activated in MCF7 mammospheres. First, western blot analysis revealed that the level of NRF2 protein was substantially higher in mammospheres than in the monolayers. NRF2 protein levels in the total cell lysate as well as in the nuclear fraction were higher in the mammospheres (Figure 2A). Accordantly, we observed that ARE-driven luciferase activity was significantly enhanced in mammospheres (Figure 2B), indicating that NRF2 activity increased in the MCF7 mammospheres. In addition, the level of the *NRF2* transcript was 1.8 times higher in the mammospheres than in the monolayers (Figure 2C). It is noteworthy that the increase in the level of NRF2 was sphere culture-specific. When MCF7 mammospheres were dissociated and re-differentiated in normal adherent plates for 3 d, the level of NRF2 protein in the total cell lysate returned to the level observed in MCF7 monolayers (Figure 2D). These results indicate the increase in NRF2 signaling is mammosphere-specific. Nonetheless, NRF2 activation could be associated with particular components in the sphere culture medium, such as growth factors. In order to exclude this possibility, we cultured MCF7 cells in a normal adherent plate with the sphere culture medium for 3 d. In these conditions, MCF7 did not form spheres (Figure 2E) and, in contrast to what was observed in the MCF7 mammosphere cultures, NRF2 and KLF4 protein levels were not elevated (Figure 2F). This also verified the mammosphere-specific NRF2 activation. In addition, we could confirm the NRF2 increase in sphere-cultured colon carcinoma HCT116 and ovarian carcinoma A2780: levels of NRF2 protein along with CSC marker KLF4 were significantly higher in spheres when compared to corresponding monolayer cells (Figure 2G). Collectively, these results indicate that NRF2 activation may be involved in the enhanced expression of efflux transporters and detoxifying/antioxidant genes in MCF7 mammospheres.

### Stabilization of NRF2 protein in MCF7 mammospheres

**Figure 3:**
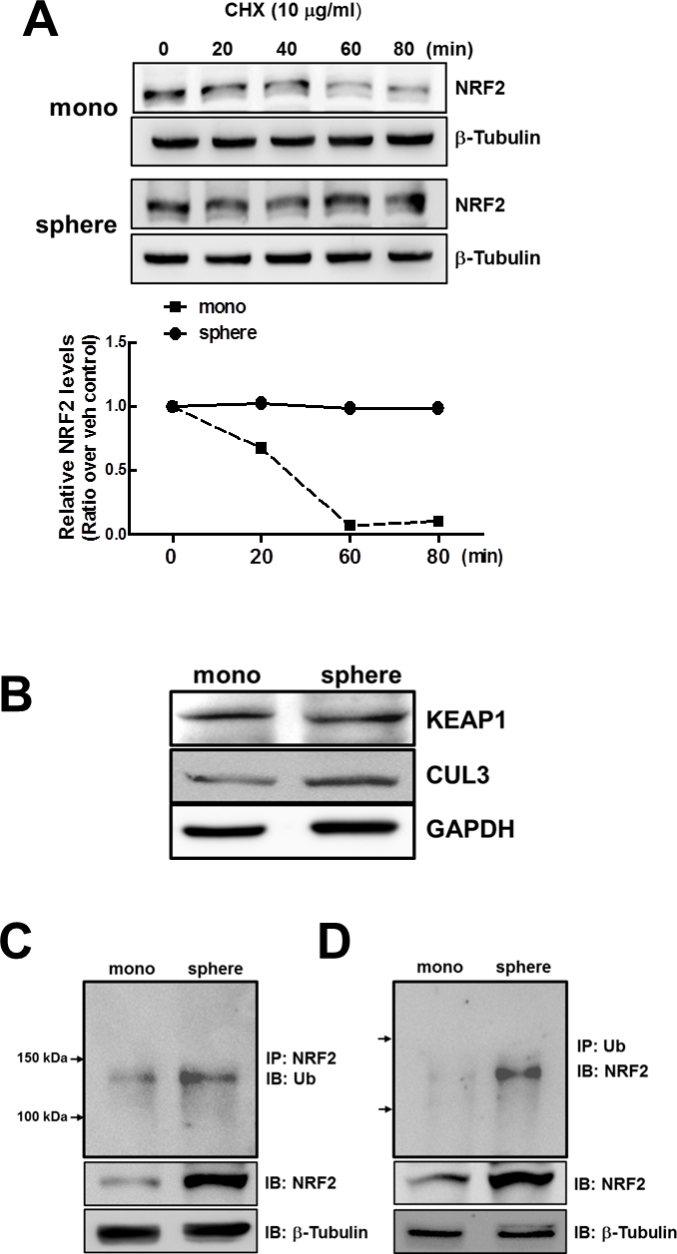
Stabilization of NRF2 protein in MCF7 mammospheres **(A)** NRF2 protein stability was assessed in monolayers and mammospheres following the treatment with protein synthesis inhibitor CHX (10 μg/mL). At each time point, cells were harvested and NRF2 protein levels were monitored by western blot analysis. A bar graph represents the relative NRF2 protein levels at each time point. **(B)** KEAP1 and CUL3 protein levels were determined in monolayers and mammospheres by western blot analysis. **(C–D)** For detection of ubiquitinated NRF2, total protein lysates of monolayers and mammospheres were immunoprecipitated with an NRF2 **(C)** or Ub antibody **(D)**, followed by immunoblot analysis with the Ub or NRF2 antibody.

In order to elucidate the mechanism involved in the increase of NRF2 protein in mammospheres, we monitored NRF2 protein stability in the presence of protein synthesis inhibitor cycloheximide (CHX). When cells were incubated with CHX (10 μg/mL) for 0–80 min, NRF2 protein levels rapidly diminished with time in MCF7 monolayers, while NRF2 protein levels did not show any decrease up to 80 min in MCF7 mammospheres (Figure 3A). These results indicate that enhanced NRF2 level in mammospheres is due to protein stabilization. Nonetheless, there was no significant difference in KEAP1 levels between monolayers and mammospheres (Figure 3B). In addition, CUL3 protein level was relatively higher in mammospheres, which does not support NRF2 stabilization.

Since NRF2 stabilization did not accompany KEAP1 change in mammospheres, we next determined ubiquitinated NRF2 levels. Endogenous NRF2 in the monolayers and mammospheres was immunoprecipitated with an NRF2 antibody and immunoblotted using an ubiquitin (Ub) antibody. The results showed that the level of ubiquitinated NRF2 was higher in the mammospheres than in the monolayers (Figure 3C). Accumulation of ubiquitinated NRF2 in mammospheres was also confirmed by Ub immunoprecipitation followed by NRF2 immunoblotting (Figure 3D). These data indicate that NRF2 protein stabilization can be associated with repressed degradation of ubiquitinated NRF2.

### Reduced proteasome function in MCF7 mammospheres

**Figure 4:**
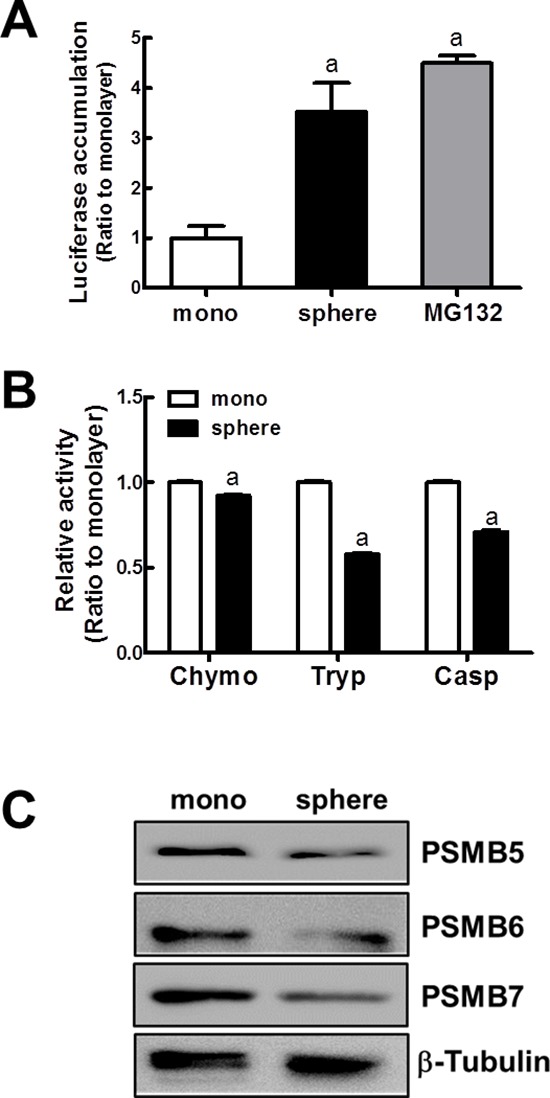
Reduced proteasome function in MCF7 mammospheres **(A)** To monitor the proteasome function, MCF7 cells in monolayers and mammospheres were transfected with the pLKO.1-ODCpdd-luciferase plasmid. The proteasome sensitive luciferase activity was then assessed. **(B)** Proteasome catalytic activities were monitored by measuring chymotrypsin-like (Chymo), trypsin-like (Tryp), and caspase-like (Casp) peptidase activities. Values represent the mean ± SE from 4–5 experiments. ^a^*P* < 0.05 compared with MCF7 monolayer. **(C)** Protein levels for PSMB5, PSMB6, and PSMB7, catalytic core subunits of the proteasome were determined by western blot analysis.

In an attempt to elucidate causative mechanisms of NRF2 accumulation, proteasome function was assessed in MCF7 mammospheres. For this, we evaluated the level of proteasome substrate accumulation using the pLKO.1-ODCpdd-luciferase plasmid. In this construct, the proteasome destruction domain (pdd) from ornithine decarboxylase (ODC) was fused to the luciferase-containing pLKO.1 plasmid. The plasmid was transfected in MCF7 to monitor the proteasome function. In mammospheres, the proteasome sensitive luciferase activity was significantly high when compared to MCF7 monolayers. The luciferase activity was similar to that of the MG132-treated monolayer group (Figure 4A), indicating that proteolytic proteasome activity is reduced in the mammospheres. Protease activity of the 26S proteasome is mediated by three catalytic subunits. The catalytic core subunits of the proteasome are PSMB5, PSMB6, and PSMB7, presenting chemotrypsin-like, caspase-like, and trypsin-like peptidase activities, respectively. When these peptidase activities were determined using fluorogenic substrates, the proteasome protease activities were reduced in mammospheres. In particular, trypsin-like and caspase-like activities were significantly low in mammospheres compared to monolayers (Figure 4B). In addition, PSMB5, PSMB6, and PSMB7 protein levels were consistently reduced in mammospheres (Figure 4C). These results indicated that reduced proteasome activity might be responsible for the enhanced ubiquitinated NRF2 accumulation in mammospheres.

### Involvement of elevated p62 protein in NRF2 activation

**Figure 5:**
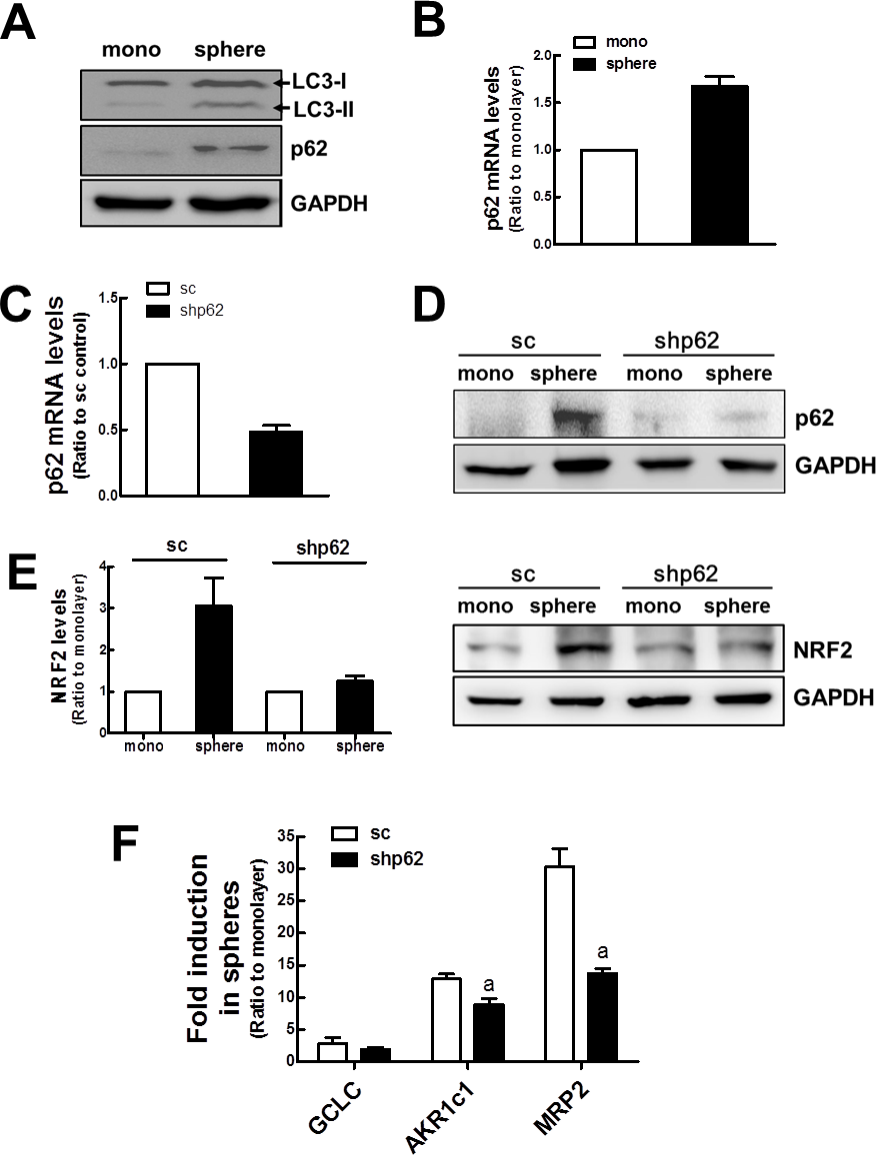
Involvement of p62 in NRF2 activation in mammospheres **(A)** LC3 (LI3-I and LC3-II) and p62 protein levels were determined in monolayers and mammospheres. **(B)** *p62* transcript levels were assessed in monolayers and mammospheres by real-time PCR. **(C)** Transcript levels for *p62* transcript levels were monitored in stable MCF7 cell lines expressing the nonspecific scRNA (sc) or p62*-*specific shRNA (shp62). Values represent the mean ± SE from 3 experiments. **(D)** p62 protein levels were determined in monolayers and mammospheres of sc and shp62 MCF7. **(E)** NRF2 protein levels were determined in sc and shp62 mammospheres. Relative NRF2 levels were quantified. **(F)** *GCLC, AKR1c1*, and *MRP2* transcript levels were assessed in sc and shp62 mammospheres by real-time PCR. Values represent the mean ± SE from 3 experiments. ^a^*P* < 0.05 compared to sc mammospheres.

Experimental evidence showed that proteasome inhibition could activate autophagy as a compensatory mechanism [32]. In our mammosphere system, the level of the ubiquitinated total proteins was not higher in MCF7 mammospheres than that in the MCF7 monolayers, although the proteasome function was reduced (Supplementary Figure S3). This may imply the compensatory activation of autophagy in mammospheres. Indeed, LC3-II protein was accumulated in the mammospheres, which reflects an increased autophagy activity (Figure 5A). In addition, both protein and mRNA levels of p62 were increased in the mammospheres (Figure 5A and 5B). Since p62 can regulate NRF2 via a non-canonical pathway [25], these data suggest that altered autophagy activity in mammospheres may be an additional contributing factor to NRF2 stabilization. To investigate a potential correlation between p62 and NRF2, we established the *p62-*knockdown stable cell line (shp62) and examined NRF2 expression in mammospheres. Our established knockdown cells displayed a 52% reduction in *p62* mRNA level when compared to the nonspecific sc control cell line (Figure 5C). After mammosphere formation, the increase in p62 was not observed in shp62 cells, confirming *p62* knockdown (Figure 5D). Importantly, we found that *p62* knockdown suppressed NRF2 increase in mammospheres. NRF2 protein levels in total cell lysates were not elevated in the *p62* knockdown group (Figure 5E). Accordantly, the increase in *AKR1c1* and *MRP2* transcript levels was significantly repressed by *p62* knockdown (Figure 5F). Taken together, these data imply that the increased p62 level in the mammosphere is also associated with NRF2 accumulation and consequent activation.

### Retarded mammosphere growth in *NRF2*-knockdown MCF7

**Figure 6:**
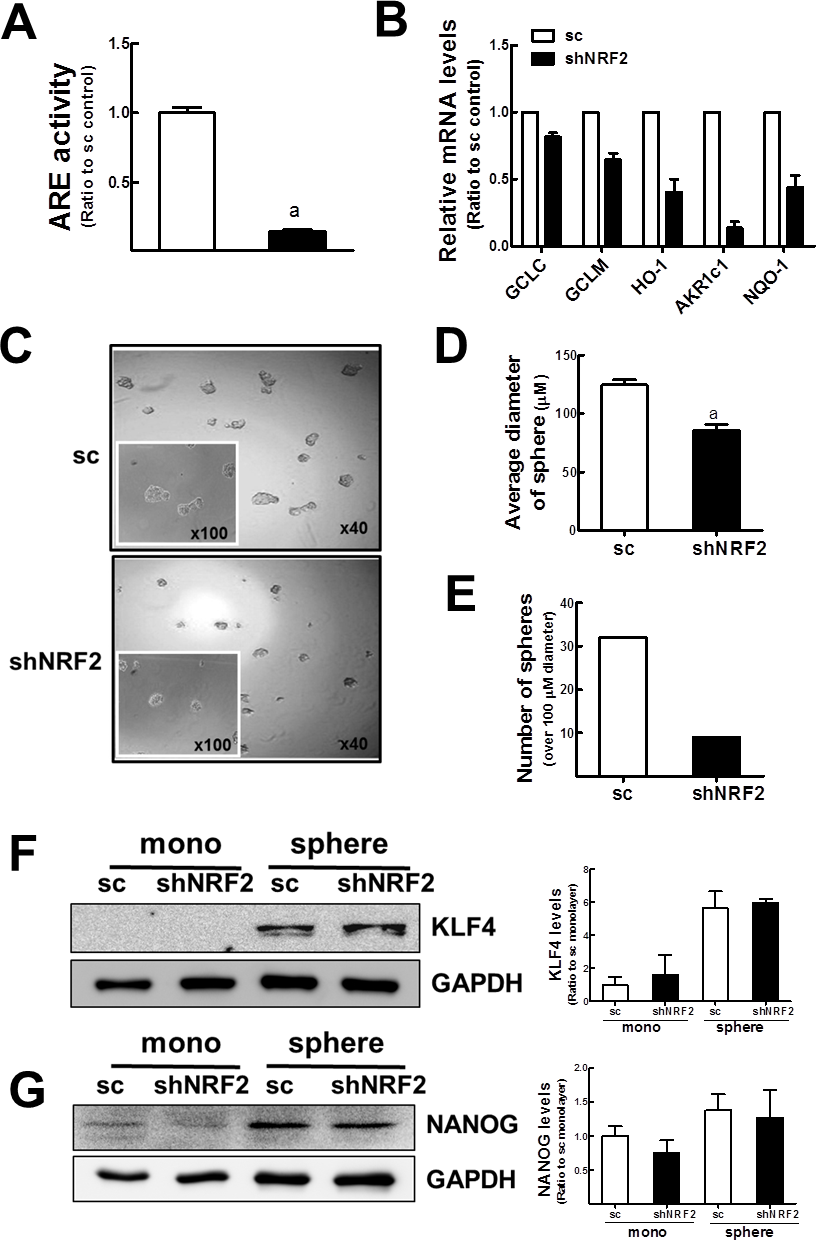
Delayed mammosphere growth in *NRF2* knockdown MCF7 **(A)** ARE-driven luciferase activity was monitored in the sc and shNRF2 cells. Values represent the mean ± SE from 3 experiments. ^a^*P* < 0.05 compared to the sc control cells. **(B)** *GCLC, GCLM, HO-1, AKR1c1,* and *NQO-1* transcript levels were assessed in the sc or shNRF2 cells by real-time PCR analysis for relative quantification. Values represent the mean ± SE from 3 experiments. **(C)** Mammosphere formation was assessed after 6 d of sc and shNRF2 MCF7 mammosphere culture. ×40 or ×100 magnification. **(D–E)** Average diameters **(D)** and number of mammospheres over 100 μm **(E)** were determined in the sc and shNRF2 groups using image processing ToupView software. Measurements were carried out among 38 spheres in the microscopic observation area. ^a^*P* < 0.05 compared to the sc mammospheres. **(F–G)** KLF4 **(F)** and NANOG **(G)** protein levels were verified in the sc and shNRF2 mammospheres by western blot analysis. Band intensities were quantified in bar graphs. Values represent the mean ± SE from 3 experiments.

In an attempt to investigate the role of NRF2 activation in mammospheres formation and CSC characteristics, we established the stable MCF7 cell line with *NRF2* knockdown (shNRF2). *NRF2* transcript level was reduced by 80% (Supplementary Figure S4) and ARE-driven luciferase activity was repressed by 85% in *NRF2* knockdown cells compared to the sc control (Figure 6A). In addition, the expression of target genes such as *GCLC, GCLM, HO-1, AKR1c1,* and *NQO-1* was decreased in shNRF2 cells (Figure 6B).

First, the involvement of NRF2 in mammosphere formation was examined by the comparison of sphere growth between the sc control and shNRF2 cells. When mammosphere formation and growth were examined 6 d after plating cells at a low density, shNRF2 mammosphere exhibited a delayed growth (Figure 6C). The average diameter of mammospheres in sc control cells was 124.5 μm, while the sphere diameter of shNRF2 mammospheres was 86 μm (Figure 6D). Similarly, among 38 spheres in the microscopic area, the number of mammospheres with over 100 μm diameter was significantly lower in the *NRF2* knockdown MCF7 (Figure 6E). These results indicate that NRF2 facilitates mammosphere formation and growth. Thus, it can be hypothesized that *NRF2* knockdown also affects CSC marker expression. To verify this hypothesis, the levels of KLF4 and NANOG, two CSC markers, were measured by immunoblot analysis. Unlike our hypothesis, there were no noticeable differences in KLF4 and NANOG expression between sc control and shNRF2 mammospheres (Figure 6F and 6G).

### Enhanced ROS and cell death in shNRF2 mammospheres

**Figure 7:**
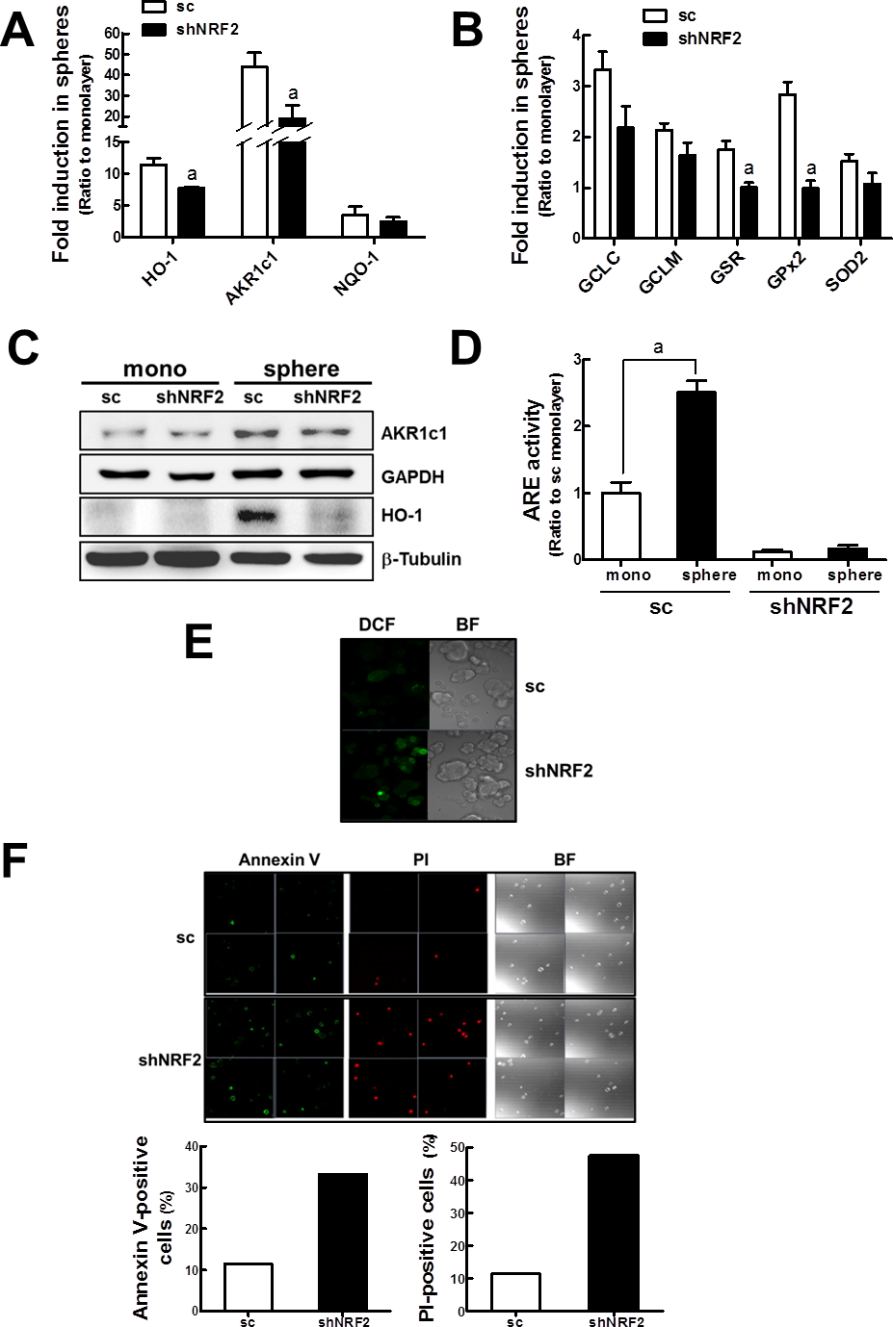
Enhanced ROS and cell death in shNRF2 mammospheres **(A)** *HO-1, AKR1c1*, and *NQO-1* transcript levels were assessed in the sc and shNRF2 mammospheres by real-time PCR. **(B)** *GCLC, GCLM, GSR, GPx2,* and *SOD2* transcript levels were monitored in the sc and shNRF2 mammospheres. Values represent the mean ± SE from 3 experiments. ^a^*P* < 0.05 compared to the sc mammospheres **(C)** AKR1c1 and HO-1 protein levels were determined in the sc and shNRF2 mammospheres. **(D)** ARE-driven luciferase activities were monitored in sc and shNRF2 monolayers and mammospheres. Values represent the mean ± SE from 3 experiments. ^a^*P* < 0.05 compared with the sc monolayers. **(E)** Intracellular ROS levels in sc and shNRF2 mammospheres were quantified using a fluorogenic dye carboxy-H_2_DCFDA. **(F)** To monitor cell death in mammospheres, suspended MCF7 from spheres were stained with an early apoptosis-specific marker Annexin V or a late apoptosis/necrosis-specific marker PI, and confocal microscopic observation was carried out. Annexin V-positive or PI-positive cell numbers are shown in the graphs.

It was apparent that NRF2 stabilization led to elevated detoxifying/antioxidant gene expression in mammospheres. This phenomenon may be an underlying mechanism of delayed mammosphere growth in the *NRF2* knockdown group. Indeed, the increase in *AKR1c1, NQO1*, and *HO-1* in mammospheres was suppressed by *NRF2* knockdown. *AKR1c1* increase in sc mammospheres was 44-fold higher than monolayers, while the value in shNRF2 mammospheres was 19-fold increase (Figure 7A). In addition, mammosphere GSH reductase (*GSR*) and ROS detoxifying *GPx* increases were repressed in shNRF2 mammospheres (Figure 7B). Similar patterns were observed in immunoblot analysis using antibodies against HO-1 and AKR1c1 (Figure 7C). Mammosphere ARE luciferase activity was also much lower in *NRF2* knockdown cells (Figure 7D).

When intracellular ROS level was visualized by confocal microscopy using a fluorogenic dye carboxy-2′,7′-dichlorofluorescein diacetate (carboxy-H_2_DCFDA), shNRF2 mammospheres exhibited a higher level of ROS than sc mammospheres (Figure 7E). Next, to monitor cell death in mammospheres, sc and shNRF2 mammospheres were dissociated into single cells and then stained with an early apoptosis-specific marker, Annexin V, or a late apoptosis/necrosis-specific marker, PI. The analysis revealed that shNRF2 mammospheres retained a greater number of Annexin V- and PI-positive cells than the sc control mammospheres (Figure 7F). These results suggested that a low expression of antioxidant genes could result in elevated ROS and enhanced cell death in *NRF2* knockdown mammospheres, further leading to delayed mammosphere growth.

### Anticancer drug sensitization of mammospheres by *NRF2* knockdown

**Figure 8:**
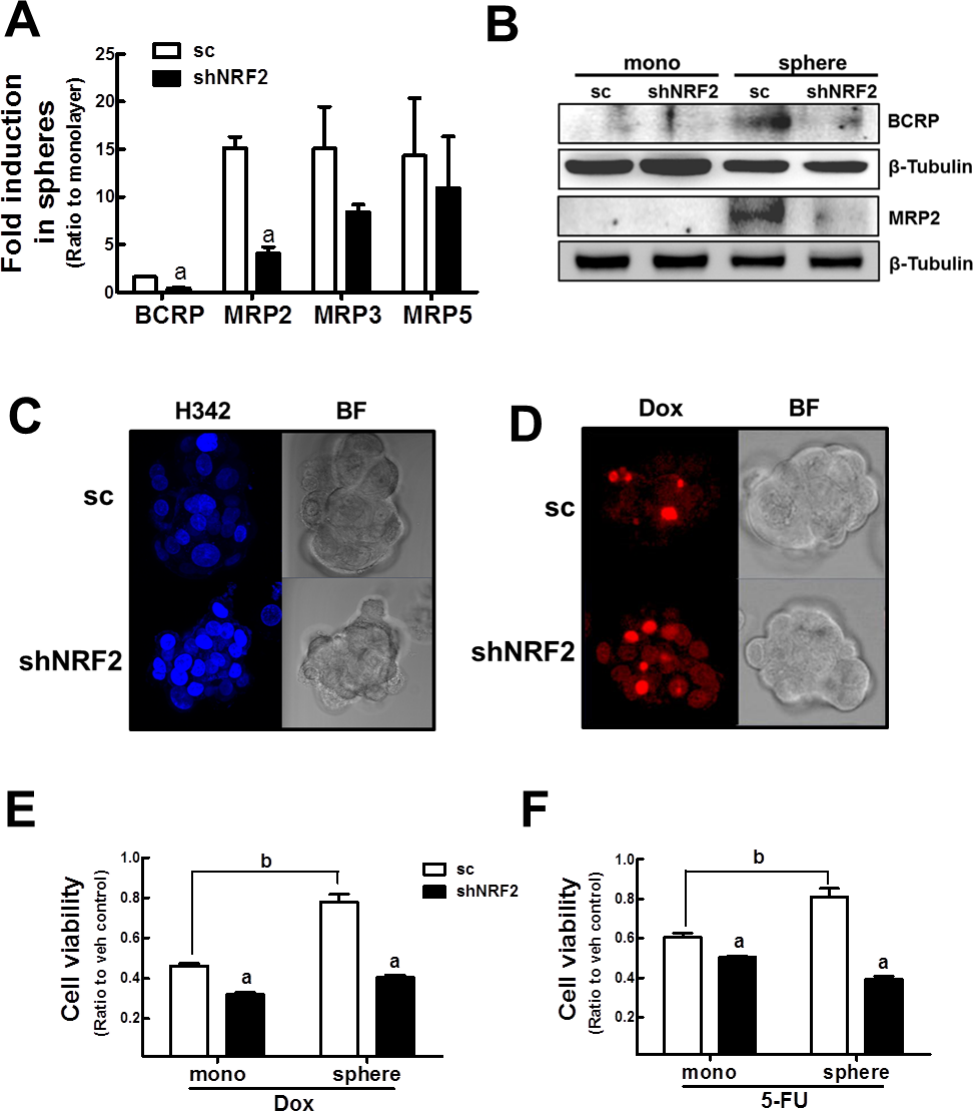
*NRF2* knockdown-mediated sensitization of mammospheres to anticancer drugs **(A)** *BCRP, MRP2, MRP3,* and *MRP5* transcript levels were assessed in the sc and shNRF2 mammospheres by real-time PCR. Values represent the mean ± SE from 3 experiments. ^a^*P* < 0.05 compared to the sc mammospheres. **(B)** BCRP and MRP2 protein levels were evaluated in the sc and shNRF2 mammospheres by western blot analysis. **(C–D)** The sc or shNRF2 mammosphere were incubated with Hoechst 33342 (H342, C) or doxorubicin (Dox, D) and fluorescent intensity was evaluated using a confocal microscopy. **(E–F)** Cell viability was monitored in the sc and shNRF2 mammospheres after doxorubicin (Dox, E) or 5-FU **(F)** treatment for 72 h. Values represent the mean ± SE from six to eight sampled wells. ^a^*P* < 0.05 compared to sc control. ^b^*P* < 0.05 compared to sc monolayers.

Mammosphere chemoresistance can be related with NRF2 activation and consequent drug efflux. In *NRF2* knockdown mammospheres, the increase in efflux transporters such as *BCRP* and *MRP2* were significantly attenuated when compared to the sc control mammospheres (Figure 8A). Repressed expression of efflux transporters was confirmed by immunoblot analysis using MRP2 and BCRP antibodies (Figure 8B). Accordingly, Hoechst 33342-resistant cells, which represent a side population of CSC, were decreased in *NRF2* knockdown mammospheres (Figure 8C). Differential transporter increases can lead to sensitization to anticancer drug treatment. Indeed, *NRF2* knockdown mammospheres accumulated higher amount of doxorubicin than sc control mammospheres (Figure 8D). In addition, 3-(4,5-dimethylthiazol-2-yl)-2, 5-diphenyltetrazolium bromide (MTT) analysis after a 72 h-doxorubicin incubation revealed that shNRF2 mammospheres did not develop doxorubicin resistance in contrast to the sc control mammospheres (Figure 8E). Similarly, the sc control MCF7 acquired 5-fluorouracil (5-FU) resistance after sphere formation, whereas shNRF2 mammospheres showed a similar response to 5-FU cytotoxicity compared to monolayers (Figure 8F). Collectively, these data suggested that mammosphere anticancer resistance, which is one of CSC characteristics, is largely mediated by NRF2 activation.

## DISCUSSION

Mounting evidence showed the correlation between NRF2 and cancer resistance. In a number of human tumors and cancer cell lines, NRF2 level increases and the resulting target gene expression confers cancer cell protection against the oxidizing microenvironment, anticancer therapy, and ionizing radiations [17]. CSCs are highly resistant to the conventional cancer therapies; therefore the existence of CSC populations in tumors is considered as the main cause of tumor relapse and aggressiveness [7, 8]. In the present study, we observed that NRF2 activity and its target genes expression were elevated in nonadherent MCF7 mammospheres, which are considered as a CSCs-enriched system. As underlying molecular mechanisms, the proteasome function was reduced and p62 level increased in mammospheres. NRF2-driven expression of detoxifying/antioxidant genes and ABC transporters in mammospheres facilitated sphere growth and anticancer drugs resistance. Notably, NRF2 increase in cultured tumor spheres could be observed in other type of cells such as colon cancer and ovarian cancer cells, indicating the universal role of NRF2 in CSCs-enriched system. These results suggest that NRF2 is implicated in the survival/growth and stress resistance of breast CSCs.

Increased expression of ABC transporters has been recognized as a representative characteristic of CSCs [33, 34]. As a molecular mechanism, the involvement of several stemness transcription factors in ABC transporters expression has been demonstrated. In chemoresistant cancer cells, a stemness transcription factor OCT4 showed a positive correlation with MDR1 and BCRP expression levels [35]. SOX2 enhanced MRP3 and MRP6 expression in patient-derived GCS, resulting in significant chemoresistance [36]. We observed that the levels of NRF2 and ABC transporters were elevated in MCF7 mammospheres, and the ABC transporters increase was significantly affected by *NRF2* knockdown, indicating the role of *NRF2* in mammosphere transporter expression. As the increase in stemness factors such as KLF4, NANOG, SOX2, and OCT4 was not affected in *NRF2* knockdown mammospheres, NRF2 decrease can be responsible for the reduced ABC transporters expression. The involvement of NRF2 in transporter expression has been demonstrated in various cell types. *MRP2* gene induction was observed in human and mouse hepatoma cells after exposure to NRF2 activators such as butylated hydroxyanisole [37]. The human *BCRP* gene promoter presents the ARE sequence and NRF2 can transactivate BCRP expression in non-small cell lung cancer cells [38]. Together with these reports, our data suggest a novel role of NRF2 in breast CSCs phenotype by elevating ABC transporters.

Intracellular redox state is one important modulator in stem cell maintenance and pluripotency. In *atm*-deficient mice, ROS level is increased in various organs due to repressed expression of catalase, superoxide dismutase (SOD), and GPx. Thus, hematopoietic stem cells (HSCs) from *atm*-deficient mice showed decreased reconstitution capacity with high ROS level [39]. HSCs population with low ROS presented higher levels of Notch1 expression and self-renewal capacity than HSCs with high ROS [40]. In addition, treatment of high ROS HSCs with an antioxidant, N-acetylcysteine (NAC), could recover HSC function. Based on these, it can be hypothesized that, similar to HSCs, CSCs in tumors may retain low ROS levels. Indeed, in the CSC population from primary human breast cancers, the expression of GSH synthesis enzymes was significantly higher than in the corresponding non-tumorigenic cells [34]. The leukemia stem cell population expressed higher GPx levels and, thereby, contained lower ROS level [41]. All together, these results suggest the importance of the ROS detoxifying system in stem cell maintenance. Thus, the potential contribution of NRF2 signaling to CSC biology could be an area of interest. We provided evidence that NRF2 signaling is elevated in CSCs-enriched mammospheres and this increase appears to play a role in ROS reduction and sphere growth enhancement. Similarly, several recent reports have demonstrated the increase of NRF2 signaling in CSCs. In alveolar basal stem cells (ABSCs), dynamic ROS increase could activate NRF2, which, in turn, stimulates Notch-1 signaling to enhance self-renewal of ABSCs [42]. A very recent study by Wu et al. showed that mammospheres have higher chemoresistance and lower ROS levels than monolayer cells, and suggested that NRF2 activation can be a cause [43]. Specifically, this study described that the treatment of mammospheres with NRF2 inhibitor could control sphere chemoresistance. On the other hand, Achuthan et al [44] demonstrated that persistent drug treatment generated stable colonies with tumor stem cell phenotypes and indicated that these cells retained low ROS levels and increased NRF2 activity [44]. In addition, this study presented the reduced proteasome activity and increased p21 as underlying signaling events in drug-induced tumor stem cells. Together with these reports, our study strengthens the role of NRF2 in CSC characteristics by using knockdown spheres and different cancer cell lines. Moreover, we elucidated that the proteasome dysfunction and p62 elevation are the driving signaling changes to elevate NRF2 in mammospheres.

The link between the proteasome and CSCs has been described in several previous reports. A study by Vlashi et al. showed that proteasome activity is low in breast and glioma cancer cell line spheres when compared to monolayer cells [45, 46]. Particularly, they showed that the cancer cell population with low proteasome activity exhibited an enhanced sphere forming capacity and increased CSCs marker expression; therefore they proposed that the low proteasome activity can be a novel marker of CSCs. As supportive clinical evidence, proteasome subunit expression in head and neck squamous cell carcinoma tissues was negatively correlated with patient survival [47]. These data suggest that the alteration of proteasome function can have a specific role in CSCs. For instance, a recent study showed that the RNA-binding protein Musashi-1 could mediate proteasome down-regulation and, consequently, Notch-1 is stabilized and CSCs signaling could be enhanced [48]. As additional evidence, our data support the role of proteasome alteration in CSC phenotypes: the decreased proteasome function contributed to stress resistance and growth facilitation of CSC-enriched cancer spheres by stabilizing NRF2. Since NRF2 activation can be achieved by the incubation of cells with proteasome inhibitors [49], accumulated ubiquitinated NRF2 and total NRF2 protein in mammospheres can activate target gene expression. On the other hand, there is a report showing a distinct phenomenon. In a study by Jang et al, NRF2 expression was high in human embryonic stem cells (hESCs), and in turn, NRF2 elevated proteasome activity by increasing the proteasome chaperone expression [50]. It was concluded that high levels of NRF2 and proteasome contributed to the self-renewal activity of hESCs; however, activated proteasome appeared to affect NRF2 level eventually, leading to NRF2 decrease upon differentiation. Overall, this study showed the involvement of dynamic interplay between proteasome and NRF2 in stemness development. Although the status of proteasome activity is not coherent, these reports support the potential importance of proteasome and NRF2 in CSCs biology.

One of the novel findings of our study would be the role of autophagy-associated p62 increase in mammospheres, and its role in CSCs phenotypes. Although the proteasome function was reduced, we found that the total level of ubiquitinated proteins was not higher in mammospheres than in monolayer cells. This implies that an alternative pathway takes charge of protein degradation. Indeed, LC3-II level, which is accepted as a marker of autophagy activity, was found to be elevated, indicating that autophagic protein degradation maintains protein homeostasis in mammospheres. Whereas, it was notable that both the transcript and protein levels of p62, a substrate of autophagy, were elevated in mammospheres. Since the regulation mechanism of p62 protein is highly complex [51], we hypothesized that p62 increase may have a specific role in cancer spheres. In particular, there have been lines of evidence indicating a positive regulation of NRF2 by p62. Overexpression of p62 led to disruption of NRF2-KEAP1 binding through the direct interaction with KEAP1, resulting in NRF2 stabilization [25, 26]. In other system, KEAP1 protein was constantly degraded by the autophagy process through the action of p62 [52]. These indicate that p62 can activate NRF2 signaling in certain conditions. In effect, a number of human tumors accumulate p62 and this phenomenon was associated with NRF2 activation [53]. Our current data adduce an additional role of p62 in CSC-enriched mammospheres, showing that p62 activates NRF2 signaling. As direct evidence, *p62* knockdown MCF7 mammospheres displayed lower levels of NRF2 and its target gene expression. The relative contribution of the proteasome and p62 to NRF2 activation remains elusive. Rather than a separate contribution, these two phenomena could be dynamically linked. For example, p62 is also a known NRF2 target gene, generating a positive feedback loop between NRF2 and p62 [24]. Our results show that *p62* mRNA level was also increased in mammospheres and this raises the possibility that the proteasome reduction-mediated NRF2 increase may cause p62 increase and a further activation of NRF2. Additionally, proteasome dysfunction is known to be a stimulator of autophagy as a compensatory mechanism. In mutant mice with reduced liver proteasome activity, autophagic activity was enhanced and p62 protein level was increased, leading to NRF2 activation [54]. Although *p62* knockdown did not affect NRF2 activation level in this study, the results suggest that proteasome reduction may lead to autophagic activation. Thus, it can be suggested that an altered proteasome function in mammospheres could lead to NRF2 stabilization and this in turn, could result in p62 elevation and additional feed forward activation of NRF2. At the same time, reduced proteasome function in mammospheres activates autophagy system and this compensation appears to maintain sphere protein homeostasis favoring specific activation of signaling molecules for sphere survival.

Collectively, our data showed the role of NRF2 increase in CSC phenotypes using the CSC-enriched cancer cell spheres. In particular, we revealed that proteasome reduction and p62 accumulation contributed to NRF2 protein stabilization and enhanced sphere growth/survival and stress resistance.

## METHODS

### Reagents

Antibodies recognizing SOX2, KLF4, NANOG, MRP2, BCRP, CUL3, p62, LC3, glyceraldehyde 3-phosphate dehydrogenase (GAPDH), and PSMB5 were from Cell Signaling Technology (Danvers, MA, USA). Antibodies against PSMB6 and PSMB7 were purchased from Enzo Life Sciences (Farmingdale, NJ, USA). NRF2, KEAP1, Ub, lamin B, and β-tubulin antibodies were obtained from Santa Cruz Biotechnology (Santa Cruz, CA, USA). The luciferase reporter plasmid containing the ARE was a gift from Dr. Wakabayashi (University of Pittsburg, PA, USA). The lentiviral expression plasmids for human *NRF2* or *p62* short hairpin RNA (shRNA), Mission^TM^ Lentiviral Packaging Mix, hexadimethrine bromide, puromycin, 5-FU, doxorubicin, CHX, and MTT were from Sigma-Aldrich (Saint Louis, MO, USA). PI was purchased from Biolegend (San Diego, CA, USA). Hoechst 33342 (H342) and carboxy-H_2_DCFDA were purchased from Life Technologies (Carlsbad, CA, USA). The SYBR premix ExTaq system was obtained from Takara (Otsu, Japan). Substrates for proteasome proteases (Suc-LLVY-AMC, Z-LLE-AMC, and Z-ARR-AMC) were from EMD Chemicals (Darmstadt, Germany).

### Cell culture

The human breast carcinoma cell line MCF7 was purchased from the American Type Culture Collection (Rockville, MD, USA). MCF7 cells were maintained in Dulbecco's modified Eagle's medium (DMEM) (HyClone, Logan, UT, USA) with 10% fetal bovine serum (FBS; HyClone) and penicillin/streptomycin (HyClone). Human colon carcinoma cell line HCT116 and ovarian carcinoma cell line A2780 were obtained from ATCC and the European Collection of Cell Cultures (Salisbury, Wiltshire, UK), respectively. The cells were grown at 37°C in a humidified 5% CO_2_ atmosphere.

### Mammosphere culture

MCF7 cells were plated at a density of 20,000 cells/mL in 100 mm ultralow attachment plates (Corning Costar Corp., Cambridge, MA, USA). Cells were grown in a serum-free DMEM and Nutrient Mixture F-12 medium supplemented with B27 (1:50, Life Technologies), 20 ng/mL epithelial growth factor (EGF), 20 ng/mL basic fibroblast growth factor (R&D System, Minneapolis, MN, USA), 5 μg/mL bovine insulin (Cell Application Inc., San Diego, CA, USA), 0.5 μg/mL hydrocortisone (Sigma-Aldrich), and penicillin/streptomycin (HyClone). In the sphere culture conditions, MCF7 cells were grown for 3 d and produced mammospheres were dissociated by incubation with 0.05% trypsin/EDTA (WelGENE Inc., Daegu, Republic of Korea) on day 4. Dissociated MCF7 cells were cultured in sphere conditions for another 3 d and were then harvested.

### Production of shRNA lentiviral particles

Lentiviral particles were produced in HEK 293T cells following the transfection of the cells with the relevant shRNA expression plasmid and Mission™ Lentiviral Packaging Mix as described previously [55]. Briefly, HEK 293T cells in Opti-MEM (Life Technologies) were transfected with 1.5 μg pLKO.1-*NRF2* shRNA, (5′-CCGGGCTCCTACTGTGATGTGAAATCTCGAGAT TTCACATCACAGTAGGA-3′) or pLKO.1-*p62* shRNA (5′-CCGGCCTCTGGGCATTGAAGTTGATCTCGAGATCAACTT-CAATGCCCAGAGGTTTTT-3′) with packaging mix using Lipofectamine 2000 (Life Technologies). As a nonspecific RNA, the pLKO.1-scrambled (sc) RNA plasmid was transfected in the control group. The next day, the medium containing the transfection complex was removed and lentiviral particles were harvested after 4 d.

### Establishment of *NRF2* or *p62* knockdown MCF7

MCF7 cells in 6-well plates were transduced with lentiviral particles containing the nonspecific pLKO.1-scRNA (sc), pLKO.1-*NRF2* shRNA (shNRF2), or pLKO.1-*p62* shRNA (shp62) in the presence of 8 μg/mL hexadimethrine bromide. Transduction was continued for 48 h and followed by a 24 h-recovery in complete medium. For the selection of stable transgene-expressing cells, puromycin (2 μg/mL) incubation was continued for up to 4 weeks.

### Total RNA extraction and RT-PCR analysis

Total RNA was isolated from the cells using the TRIzol reagent (Life Technologies). For the synthesis of cDNA, RT reactions were performed by incubating 200 ng of total RNA with a reaction mixture containing 0.5 μg/μL oligo dT_12–18_ and 200 U/μL moloney murine leukemia virus RT (Life Technologies). Real-time RT-PCR was carried out using a Roche Light Cycler (Mannheim, Germany) with the Takara SYBR Premix ExTaq System for relative quantification. Primers were synthesized by Bioneer (Daejeon, Republic of Korea) and primer sequences for the human genes are described in our previous studies [56, 57]. PCR primers for *CD44 are* 5′-AGCAGCACTTCAGGAGGTTAC-3′ and 5′-TGCCTCTTGGTTGCTGTCTC-3′.

### MTT assay

Viable cell numbers were determined by MTT assay. MCF7 cells were seeded at a density of 5 × 10^3^ cells/well in 96-well plates and incubated with the relevant compounds (doxorubicin, H2O2, or 5-FU) for the indicated times. MTT solution (2 mg/mL) was added to the cells and further incubated for 4 h. The MTT solution was removed, 100 μL/well of DMSO was added, and the absorbance was measured at 540 nm using a SPECTRO Star^Nano^ (BMG LABTECH GmbH, Allmendgruen, Ortenberg, Germany).

### Nuclear protein extraction

Cells were lysed with homogenization buffer (2 M sucrose, 1 M HEPES, 2 M MgCl_2_, 2 M KCl, 30% glycerol, 0.5 M EDTA, 1 M dithiothreitol, protease inhibitor cocktail, and 10% NP-40) and followed by centrifugation at 12000 *g* for 15 min to collect crude nuclear fractions. Nuclear proteins were then extracted by incubating crude nuclear fractions with extraction buffer (20 mM HEPES (pH 7.9), 1.5 mM MgCl_2_, 420 mM NaCl, 10% glycerol, 0.2 mM EDTA, and protease inhibitor cocktail) for 30 min on ice.

### Immunoprecipitation and immunoblot analysis

Cells were lysed with radioimmunoprecipitation assay (RIPA) lysis buffer (50 mM Tris [pH 7.4], 150 mM NaCl, 1 mM EDTA, and 1% NP40) containing a protease inhibitor cocktail (Sigma–Aldrich). The protein concentration was determined using a bicinchoninic acid assay (BCA) kit (Thermo Scientific, Middletown, VA, USA). Cell lysates were immunoprecipitated with anti-Ub antibody or anti-NRF2 antibody at 4°C overnight. The immune complexes were then incubated with protein A-sepharose (GE Healthcare Life Sciences, Piscataway, NJ, USA) at 4°C for 2 h, washed three times with RIPA buffer and boiled for 5 min in sample loading buffer (200 mM Tris [pH 6.8], 400 mM DTT, 8% SDS, 0.08% bromophenol blue, and 40% glycerol). The protein samples were separated by electrophoresis on 6–12% SDS-polyacrylamide gels and transferred to nitrocellulose membranes (Whatman GmbH, Dassel, Germany) using a Trans-Blot Semi-Dry Cell (Bio-Rad, Hercules, CA, USA). The membrane was blocked with 5% skimmed milk for 1 h, and then incubated with the antibodies. Following the addition of the enhanced chemiluminescence reagent (Thermo Scientific), images were detected using a GE Healthcare LAS*-*4000 mini imager (GE Healthcare Life Sciences).

### Measurement of ROS

Cellular ROS levels were determined using the fluorescent carboxy-H_2_DCFDA. Briefly, MCF7 mammospheres were incubated with 30 μM carboxy-H_2_DCFDA for 30 min and fluorescence images were obtained using appropriate filters (488/524 nm) in a LSM 710 confocal microscope (Carl Zeiss, Jena, Germany).

### Measurement of ARE-luciferase activity

The cells were seeded in 24-well plates at a density of 2.0 × 10^4^ cells/well and grown overnight. The next day, the transfection complex containing 0.5 μg of the ARE-luciferase plasmid along with 0.05 μg of pRLtk control plasmid (Promega, Madison, WI, USA) and the transfection reagent (WelGENE Inc.) was added to each well. After 18 h, the transfection complex was removed and the cells were incubated in a complete medium for 24 h. The cells were then lysed. Renilla and Firefly luciferase levels were measured using the Dual Luciferase Assay System (Promega) as described previously [58].

### Annexin V-PI staining

For the assessment of apoptotic cells in mammospheres, Annexin V (for early apoptosis) and PI (for late apoptosis) were incubated in cells and fluorescent intensities were monitored. MCF7 mammospheres were collected by gentle centrifugation and were dissociated into single cells using trypsin. After washing with cold PBS, cells were incubated with fluorescein isothiocyanate (FITC)-conjugated annexin V antibody and PI at room temperature for 10 min. Fluorescence images were then obtained using a LSM 710 confocal microscope (Carl Zeiss).

### Measurement of the proteasome proteolytic activity

Proteasome proteolytic activity was determined by measuring luciferase activities. The proteasome destruction domain (pdd) from ornithine carboxylase (ODC) [59] was fused to the luciferase-coding region and was then inserted into pLKO.1 plasmid (Addgene, Cambridge, MA, USA) to generate the pLKO.1-ODCpdd-luciferase plasmid. For the measurement of the proteasome proteolytic activity, the pLKO.1-ODCpdd-luciferase plasmid was transfected into cells and luciferase activity was monitored 24 h after transfection.

### Measurement of the proteasome peptidase activity

The peptidase activities of the 19S proteasome catalytic subunits were measured by mixing cell lysates with 50 μM of fluorogenic substrates Suc-LLVY-AMC (chymotrypsin-like proteases; PSMB5), Z-LLE-AMC (caspase-like proteases; PSMB6), or Z-ARR-AMC (trypsin-like proteases; PSMB7) in a final volume of 100 μL of reaction buffer (50 mM Tris–HCl, pH 7.8, 20 mM KCl, 5 mM MgCl_2_, and 1 mM DTT). The mixture was incubated at 37°C for 20 min and the reaction was then stopped by addition of an equal volume of 125 mM sodium borate buffer (pH 9.0) containing 7.5% ethanol. The fluorescence of the cleaved AMC moiety was measured at an excitation wavelength of 360 nm and emission wavelength of 460 nm using a SpectraMax M5 (Molecular Devices, Sunnyvale, CA, USA).

### Statistical analysis

Statistical significance was analyzed using Student's *t*-test or a one-way analysis of variance (two-way ANOVA) followed by the Student Newman–Keuls test for multiple comparisons using Prism software (GraphPad Prism, La Jolla, CA, USA).

## SUPPLEMENTARY FIGURES


