# Supplementary material for: Activation of NRF2 by p62 and proteasome reduction in sphere-forming breast carcinoma cells
Source: Oncotarget. 2015 Feb 27;6(10):8167–84. doi: 10.18632/oncotarget.3047 (PMC4480743; doi:10.18632/oncotarget.3047)
Supplement: Supplementary file 1 [file oncotarget-06-8167-s001.pdf]

SUPPLEMENTARY FIGURES

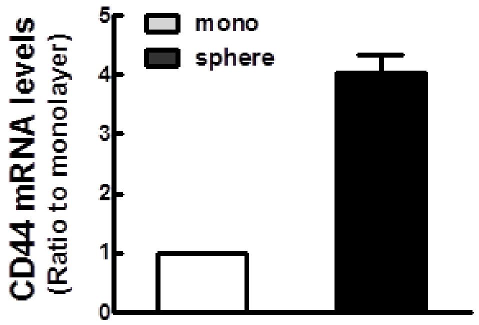

Supplementary Figure S1: Elevation of CSCs marker CD44 in MCF7 mammospheres. *CD44* transcript levels were assessed in MCF7 mammospheres using real-time PCR for relative quantification. Values represent the mean ± SE from 3 experiments.

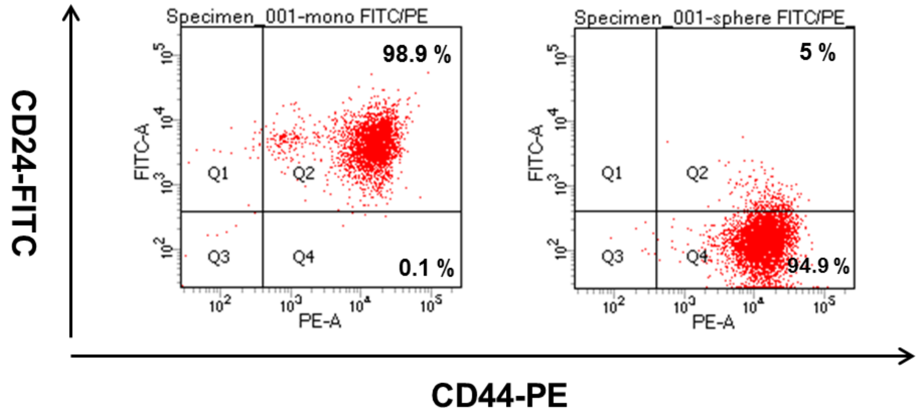

Supplementary Figure S2: Enrichment of CSCs in MCF7 mammospheres. Percentage of CD44^{+}/CD24^{-} cells in MCF7 mammospheres were quantified by flow cytometry.

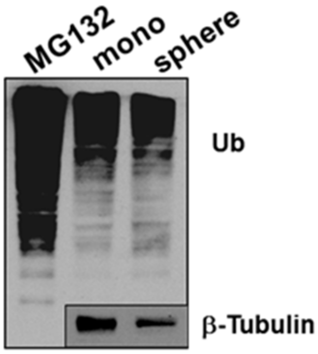

Supplementary Figure S3: Ubiquitinated protein levels in monolayers and mammospheres were determined using western blot analysis using an Ub antibody.

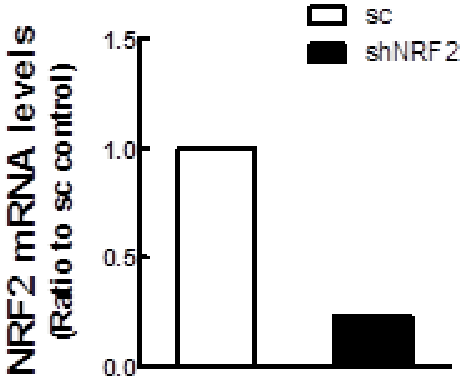

Supplementary Figure S4: *NRF2* transcript level was assessed in MCF7 cell lines expressing nonspecific scRNA (sc) or NRF2-specific shRNA (shNRF2).
